# Selection and Evaluation of Appropriate Reference Genes for RT-qPCR Normalization of* Volvariella volvacea* Gene Expression under Different Conditions

**DOI:** 10.1155/2018/6125706

**Published:** 2018-07-09

**Authors:** Jiang Qian, Yingnv Gao, Ying Wáng, Yingying Wu, Ying Wāng, Yucheng Zhao, Hongyu Chen, Dapeng Bao, Jiyang Xu, Xiaohong Bian

**Affiliations:** ^1^School of Life Science and Technology, China Pharmaceutical University, No. 24, Tongjiaxiang, Gulou District, Nanjing 210009, China; ^2^National Engineering Research Center of Edible Fungi and Key Laboratory of Applied Mycological Resources and Utilization, Ministry of Agriculture and Shanghai Key Laboratory of Agricultural Genetics and Breeding and Institute of Edible Fungi, Shanghai Academy of Agriculture Science, Shanghai, China; ^3^Jiangsu Key Laboratory of Bioactive Natural Product Research and State Key Laboratory of Natural Medicines, China Pharmaceutical University, Nanjing, Jiangsu, China

## Abstract

*Volvariella volvacea (V. volvacea)*, commonly referred to as Chinese (paddy straw) mushroom, is a basidiomycete with a protein-rich volva and pileus. Selecting appropriate reference genes is a crucial step in the normalization of quantitative real-time PCR data. Therefore, 12 candidate reference genes were selected from the* V. volvacea* transcriptome based on previous studies and then BestKeeper, geNorm, and NormFinder were used to identify reference genes stably expressed during different developmental stages and conditions. Of the 12 candidate reference genes, SPRY domain protein (*SPRYp*), alpha-tubulin (*TUBα*), cyclophilin (*CYP*), L-asparaginase (*L-asp*), and MSF1-domain-containing protein (*MSF1*) were the most stably expressed under different experimental conditions, while 18S ribosomal RNA (*18S*), 28S ribosomal RNA (*28S*), and beta-actin (*ACTB*) were the least stably expressed. This investigation not only revealed potential factors influencing the suitability of reference genes, but also identified optimal reference genes from a pool of candidate genes under a wide range of conditions.

## 1. Introduction

Quantitative real-time PCR (RT-qPCR) has emerged as a powerful and popular tool used for rapid and accurate assessment of changes in gene expression [1–3]. The reliability of gene expression measurements by RT-qPCR is strongly affected by technical factors, including template RNA quality, efficiency of complementary DNA (cDNA) synthesis, performance of primers, and normalization [4–6]. When normalizing target gene expression, selecting a stable reference gene is extremely important, especially for samples with fluctuating expression levels [7, 8]. The use of unsuitable reference genes in RT-qPCR analysis has yielded unreliable and confusing results [9, 10]. Certain reports have supported merging expression of at least three reference genes when normalizing RT-qPCR results [11, 12]. In addition, the same reference genes often cannot be used for different tissues and cells, even when the samples are derived from the same species. Therefore, selection of suitable reference genes based on a given experimental design or species is necessary [13, 14].

*Volvariella volvacea* (Bull.) Singer, i.e., straw mushroom or Chinese mushroom, is the world's third largest edible fungus and is a tropical and subtropical saprophytic fungus in the* Pluteaceae* family and* Basidiomycota* phylum [15–17]. This mushroom is an important healthy food source and valuable supplement with dietary and medicinal attributes due to being rich in certain nutrients, including proteins, vitamins, fats, and amino acids [18].* V. volvacea* is the fastest growing species of edible fungi, requiring only 7 to 12 days from sowing to fruiting and 30 days to cultivation. In addition, it has a high economic efficiency owing to only requiring a simple planting method and ample availability of raw materials [17, 19]. Filamentous fungi are important organisms frequently studied by RT-qPCR; however, identification of suitable reference genes for RT-qPCR of fungal species has received little attention. Published reports on fungal internal control genes have mainly focused on relative expression stability [20–22] and failed to evaluate absolute expression levels. Moreover, these studies have mostly involved traditional housekeeping genes in* Ascomycetes *[20, 21, 23–25], with the exception of studies on* Phakopsora pachyrhizi *[26, 27] and* Pleurotus ostreatus *[28]. Little information is currently available on* Basidiomycetes* reference genes.

Traditionally, 18S ribosomal RNA (*18S*), 28S ribosomal RNA (*28S*), *β*-actin (*ACTB*), cyclophilin (*CYP*), tubulin (*TUBα* and* TUBβ1*), glyceraldehyde-3-phosphate dehydrogenase (*GAPDH*), and ubiquitin (*UBQ*), genes with housekeeping roles in basic cellular processes, have been used as reference genes [29, 30]. However, the stability of these housekeeping genes can restrict experimental design or the use of certain treatments. In addition, different materials tend to have different genes that are stable and expression levels may differ under different experimental conditions [30, 31].

In this study, we selected 12 candidate reference genes based on* V. volvacea* transcriptome RNA-seq datasets. The expression of these candidate reference genes following different treatments was profiled. The stability of expression of these genes was further validated using RT-qPCR and statistical algorithms, including geNorm, NormFinder, and BestKeeper. Comprehensive ranking of the stability of these reference genes under each specific experimental condition was also performed.

## 2. Materials and Methods

### 2.1. Sample Preparation and Treatment

*V. volvacea* homokaryon PYd15 (ACCC52631) was obtained from the Shanghai Academy of Agricultural Science and maintained on potato dextrose agar at 32°C with periodic transfers. Mycelial samples of this strain were cultivated in potato dextrose medium with shaking at 150 rpm at 32°C and incubated in the absence or presence of NaCl, CuSO4, H_2_O_2_, HCl, NaOH, heat, or cold for four days. For fruiting body production, solid cultures of the strain were cultivated on rice straw compost as described by Chen et al. [32]. Fruiting body samples were harvested at the primordium and fruiting developmental stages according to Tao et al. [29]. The entire fruiting body was harvested, chopped, and then mixed. Each sample was prepared using a mixture of multiple fruiting bodies. All samples were immediately frozen in liquid nitrogen and then RNA was extracted. Three independent biological replicates were tested for each sample and all samples in each biological replicate were harvested from a newly produced batch.

### 2.2. Isolation of Total RNA and cDNA Synthesis

Total RNA was extracted from samples using an RNAprep Pure Plant Kit (Tiangen Biotech, Beijing, China), treated with DNase I (Ambion, USA) to digest contaminating DNA, and then purified according to the manufacturer's protocol. The integrity of the RNA was verified by electrophoresis on 1.5% (w/v) agarose gels and the quantity and quality of the RNA were measured using a NanoDrop 2000 Spectrophotometer (NanoDrop Technologies, Thermo Scientific, USA). Only RNA samples with absorption ratios of A260/280 ranging from 1.8 to 2.2 and A260/230 >1.8 were used for cDNA synthesis.

The cDNA was synthesized from 1 *μ*g total RNA in a final volume of 20 *μ*L using the PrimeScriptTM RT reagent Kit with gDNA Eraser (TaKaRa Bio Inc., Dalian, China) according to the manufacturer's instructions and then diluted 10-fold with nuclease-free water for RT-qPCR.

### 2.3. Selection and Validation of Candidate Reference Genes and Primer Design

Based on previous studies, the expression stability of the 12 candidate genes* ACTB, CYP, GAPDH, TUBα, TUBβ1, UBQ, MSF1, SPRYp, L-asp, MAPK, 18S, *and* 28S*, described in Table 1, was assessed to identify the most stable* V. volvacea* reference genes under different conditions. The primers were designed using Primer Premier 5.0 based on the following criteria: primer length of 20-27 bp, GC content of 45-55%, melting temperature ranging from 55 to 60°C, and amplicon length of 100-250 bp.

### 2.4. Amplification by RT-qPCR

Gene expression levels were examined by RT-qPCR on an Applied Biosystems 7500 Real-Time PCR system. Each reaction mixture contained 2 *μ*l prepared cDNA template, 0.4 *μ*l each forward, and reverse primers (10 nM), 6.8 *μ*l of ddH_2_O, 0.4 *μ*l ROX, and 10 *μ*l of Power SYBR Green PCR Master Mix (Life Technologies, USA) in a final volume of 20 *μ*l. Amplification cycles involved an initial denaturation step at 95°C for 5 min, followed by 40 cycles of 95°C for 15 s and 60°C for 1 min. A temperature ramp step with an initial temperature of 60°C and final temperature of 95°C was performed following the amplification for dissociation analysis. Each biological sample was tested in triplicate.

### 2.5. Gene Expression Stability Analysis

To analyze the expression stability of candidate reference genes, geNorm [33], NormFinder [34], and BestKeeper [35] were used based on the experimental design and manufacturers' instructions. For geNorm and NormFinder analysis, the raw Cp values were transformed into relative quantities using the formula 2^-ΔCT^ (ΔCT = each corresponding Ct value - same gene's lowest Ct value in different samples, where Cp is an alternative designation for Ct). These values were imported into geNorm to obtain a gene expression stability value (M). Similar to geNorm, NormFinder was used to further investigate the expression stability values (M) for each gene and the pairwise variation (V) of that gene against other reference genes was evaluated. The reference gene with the highest M was considered to have the most unstable expression, while the lowest M indicated the most stable expression. BestKeeper analysis used the untransformed Cps, the coefficients of variance (CVs), and the standard deviations (SDs) of the Cps to evaluate the stability of the reference genes. BestKeeper was also used to rank candidate expression from the most to least stable. By combining these three types of Microsoft Excel-based software, the expression stability of the candidate reference genes under different conditions was easily ranked.

### 2.6. Statistical Analysis

The RT-qPCR data was obtained from three biological replicates tested in triplicate. Unless indicated otherwise, data are presented as mean ± standard error of the mean. Statistical analyses were performed using Student's t-test. Graphs were generated using GraphPad Prism 6 (GraphPad Software, Inc., La Jolla, CA, USA). Data analysis was performed using geNorm [33], NormFinder [34], and BestKeeper [35] according to the manufacturers' instructions.

## 3. Results

### 3.1. Selection of Candidate Reference Genes, Specificity of Amplification, and PCR Efficiency

Gene names, descriptions, accession numbers, primer sequences, PCR product lengths, PCR efficiencies, and regression coefficients for the 12 candidate genes are listed in Table 1. The gene sequences of beta-actin (*ACTB*), cyclophilin (*CYP*), glyceraldehyde-3-phosphate dehydrogenase (*GAPDH*), alpha-tubulin (*TUBα*), beta-tubulin 1 (*TUBβ1*), ubiquitin (*UBQ*), MSF1-domain-containing protein (*MSF1*), SPRY domain protein (*SPRYp*), L-asparaginase (*L-asp*), mitogen-activated protein kinase (*MAPK*), and 18S (*18S*) and 28S ribosomal RNA (*28S*) were identified in the* V. volvacea* genome and confirmed by NCBI BLAST. The expression stability of these genes was assessed under various conditions, including in the presence of abiotic stresses (NaCl, CuSO_4_, H_2_O_2_, HCl, NaOH, heat, and cold) and different developmental stages. To calculate the amplification efficiency, standard curves were generated for the candidate genes using 10-fold serial dilutions of plasmid DNA containing the given genes. Based on the slopes of the standard curves (Fig.  S3), PCR efficiencies (E) and regression coefficients (R2) were calculated and are listed in Table 1 and Fig.  S3, respectively. Briefly, the R^2^ for all primers was >0.99 and the E ranged from 97.636% to 128.813%.

## 4. Expression Profiles of the Candidate Reference Genes

**Figure 1:**
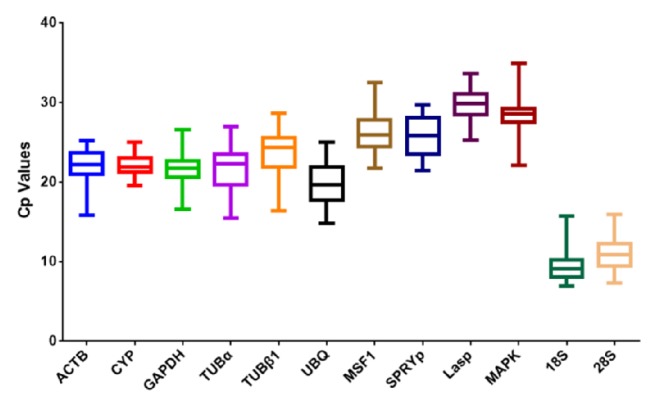
**Comparison of transcript abundances of the 12 candidate reference genes.** Boxes indicate the 25th/75th percentiles, lines represent the median, and error bars represent the maximum and minimum Cp values. The 12 candidate reference genes are listed on the x-axis.

To evaluate the stability of the reference genes in all experimental samples, the transcript abundances of the 12 candidate reference genes were measured based on their mean cycle threshold values (Cps). The mean Cps ranged from 9 to 29 and most were between 18 and 23. Across all samples,* 18S* was the most abundantly expressed gene with the lowest average Cp (9.37±1.82), followed by* 28S* (10.96±2.04),* UBQ* (19.82±2.75),* TUBα* (21.53±3.17),* GAPDH* (21.69±2.16),* ACTB* (21.89±2.35),* CYP* (22.13±1.43),* TUBβ1* (23.55±3.30),* SPRYp* (25.73±2.47),* MSF1* (26.10±2.21),* MAPK* (27.95±2.82), and finally* L-asp* (29.56±2.17). These Cps, as well as gene expression variation, are presented in Figure 1 using box-plots. Larger Cp SDs indicate more variable expression.* CYP* displayed the least variation in gene expression (22.13±1.43), indicating that it is stably expressed under different conditions and could be the optimal reference gene. Meanwhile,* MAPK *had Cps ranging from 22.12 to 33.88 and should be avoided as a reference gene. In general, the Cps in box-plot form displayed the expression profiles of the reference genes and gave us a glimpse into gene stability. However, considering the complex surroundings of edible fungi, the stability of reference genes under different conditions needs to be investigated systematically (Figure 1).

### 4.1. Expression Stability of Candidate Reference Genes

In order to further evaluate the expression stability of candidate reference genes,* V. volvacea* was exposed to different stresses (salt, oxidative, heavy metal, acid-base, and temperature stresses) or collected at different developmental growth stages. Gene expression was evaluated in these samples (three biological and technical replicates for a total of 972 Cps) using three Excel-based programs, geNorm [33], NormFinder [34], and BestKeeper [35].

### 4.2. Analysis Using geNorm

**Figure 2:**
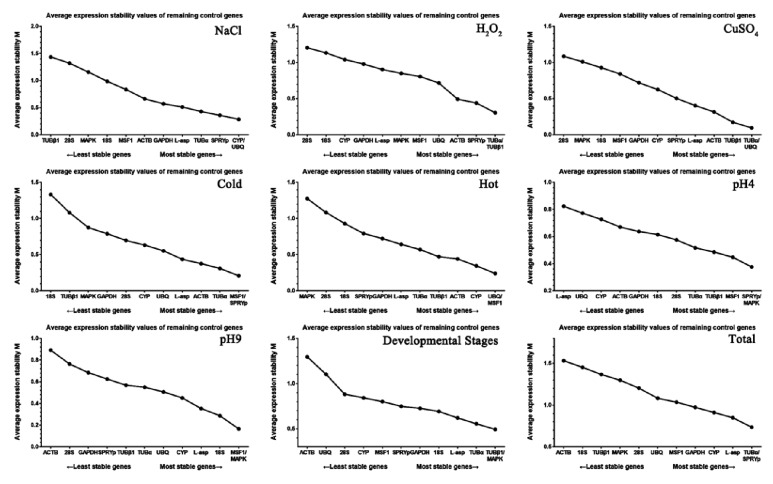
**Expression stability of 12* V. volvacea* candidate genes as predicted by geNorm analysis.** Average expression stability (M) for each condition was calculated. The least stable gene with the highest M value is on the left, while the most stable gene is on the right. The treatments and group classifications are indicated in the figure.

Analysis with geNorm measures reference gene expression stability (M) by calculating the pairwise variation for each reference gene against all other control genes and the SD of the logarithmically transformed expression ratios, where a high M means low stability [33]. For geNorm analysis, the Cps collected from the samples described above were processed on a linear scale using the ΔCp method [33]. As shown in Figure 2, different reference genes had different stabilities. The top two reference genes for RT-qPCR normalization were* TUBα* and* UBQ* for salt stress,* TUBα* and* TUBβ1* for oxidative stress,* CYP* and* UBQ* for heavy metal stress,* MSF1* and* SPRYp* for cold stress,* UBQ* and* MSF1* for heat stress,* SPRYp* and* MAPK* for acid stress,* MSF1* and* MAPK* for alkali stress, and* TUBβ1* and* MAPK* for different developmental stages. Across all samples,* TUBα* and* SPRYp* were the most stably expressed genes (Figure 2). Therefore, these two reference genes were deemed the best reference genes for the widest range of test conditions based on this present study.

### 4.3. NormFinder Analysis

NormFinder is an algorithm used to identify the optimal normalization gene in a given experimental design. Similar to geNorm, RT-qPCR data was first transformed [34]. The gene stabilities calculated using NormFinder are presented in Table 2 with gradually decreasing stabilities presented going from the top to the bottom in ranking order.* UBQ*,* SPRYp*,* MSF1*,* 18S,* and* L-asp* were the most stable reference genes in the presence of NaCl (as well as CuSO_4_ and heat), H_2_O_2_, cold (as well as acid), alkalinity, and during different developmental stages, respectively. Among the most stable reference genes,* 18S *had the lowest value and therefore could be considered the optimal reference gene. For all samples as a whole,* SPRYp* had the most stable expression. Interestingly,* UBQ* ranked near the top for 3 out of 8 tested conditions, similar to the outcomes of geNorm analysis (Figure 2). However, there were also slight differences between the geNorm and NormFinder results. For instance,* L-asp*,* CYP,* and* MSF1* were the third, fourth, and sixth most stable reference genes in geNorm (Figure 2), but the fourth, fifth, and third in NormFinder (Table 2), respectively. Therefore, an additional method of analysis was utilized to mediate these differences.

### 4.4. BestKeeper Analysis

**Figure 3:**
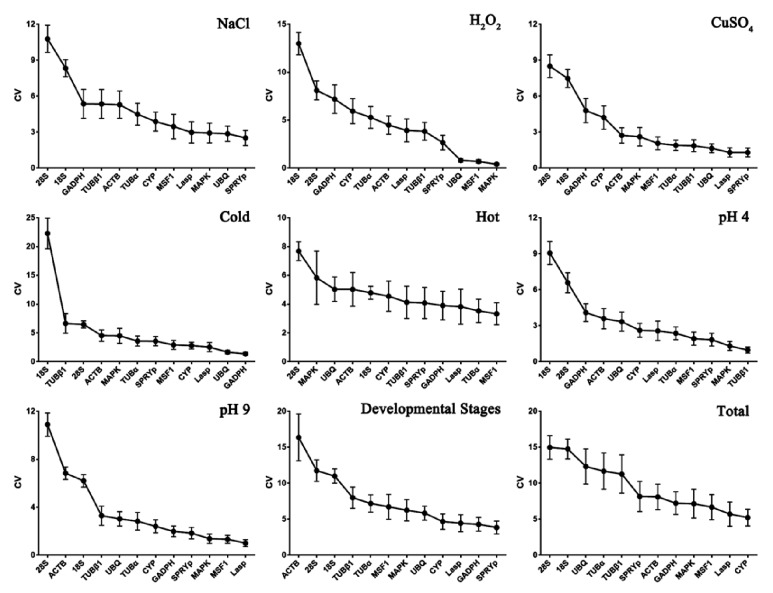
**Expression stability of 12* V. volvacea* reference genes as calculated by BestKepper.** The CVs and SDs of the candidate reference genes were used to evaluate the stability of the candidate reference genes in all tested conditions. The gene with the lowest CV and SD was considered the most stably expressed, which is on the right, while the least stable gene is on the left. The treatments and group classifications are indicated in the figure.

BestKeeper is an Excel-based tool that uses pairwise correlations to determine the stability of housekeeping genes, differentially regulated target genes, and sample integrity [35]. The CVs and SDs of the candidate reference genes were used to evaluate the stability of the candidate reference genes in all tested conditions. The gene with the lowest CV and SD was considered the most stably expressed [36]. This method differs from the geNorm and NormFinder analysis as it uses raw Cps for analysis. Similar to the results of NormFinder analysis, the CV±SD rank of the candidate genes increased gradually, suggesting the stability decreased gradually. For example,* MAPK* had a CV±SD value of 0.41±0.12 and was the most stable gene under H_2_O_2_-induced oxidative stress, while* 18S* was the least stable gene with a CV±SD of 12.98±1.15 (Figure 3). An SD>1 was considered inconsistent and any such values should be excluded [14]; therefore, none of the reference genes could be used under all conditions, as the lowest SD in this scenario was 1.16. Fortunately, in another 8 groups or experimental conditions, nearly all SD values were below 1.16, except for the most unstable one. Certain reference genes, namely,* SPRYp*,* MAPK,* and* L-asp*, had a tendency to be the most stable and were ranked among the top 3 reference genes. By contrast,* 18S* and* 28S* did not appear to be good reference genes.

## 5. Comprehensive Stability Analysis of Reference Genes 

To obtain a consensus result of the most stable reference genes as recommended by the three methods, the geometric mean of three algorithms corresponding rankings for each candidate gene was calculated (Table 3 ).* SPRYp*,* TUBα*,* CYP*,* L-asp*, and* MSF1* were ranked as the top five stable reference genes in the all samples stress;* MSF1* also comprehensively ranked first in the Cold and Hot stress subset. In H_2_O_2_ stress subset,* TUBβ1* was stably expressed most. For both the NaCl stress subset and the CuSO_4_ stress subset,* UBQ* was the most stable gene. Additionally, under acid treatment,* MAPK* was the best reference gene. The expression of* L-asp* was extremely stable under alkali stress and different developmental stages.* 18S* and* 28S* were unstably expressed in the majority of tested subsets. Owing to the geometric mean of three algorithms corresponding rankings, the results were more intuitive.

### 5.1. Optimal Number of Reference Genes for Accurate Normalization

**Figure 4:**
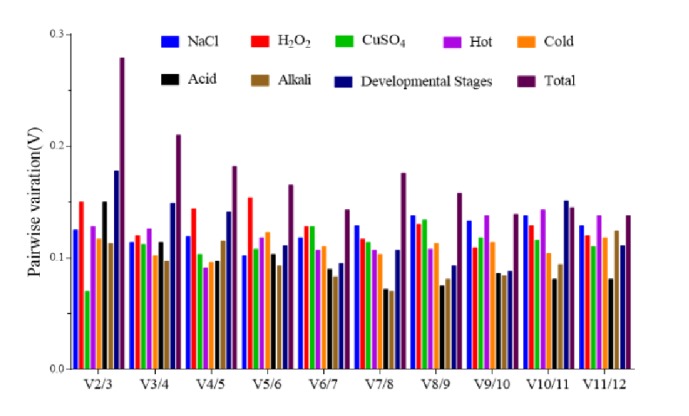
**Determination of the optimal number of reference genes for normalization by pairwise variation using geNorm.** Pairwise variation (Vn/n+1) analysis of the normalization factors (NFn and NFn+1) was performed for all samples. Different conditions are included and marked in square frames with different colors. “Total group” refers to all samples. V is the variation value, where >0.15 indicates that an additional reference gene does not improve normalization.

In addition to using average pairwise expression ratios (M) to evaluate gene expression stability, geNorm can also be used to determine the optimal number of reference genes for normalization, where pairwise variation (Vn/Vn+1) between the normalization factors is calculated for all samples and 0.15 is the proposed cut-off [33]. Based on this, the pairwise variations were calculated and are listed in Figure 4. As indicated, the two most stable reference genes were sufficient for reliable normalization under all conditions, except during different developmental stages, and an additional reference gene was unnecessary. However, three genes were necessary for normalization when evaluating different developmental stages because V2/3>0.15. While including a third reference gene may increase the credibility of RT-qPCR analysis, the proposed 0.15 value should not be considered a strict cut-off in most cases, because using a combination of the two best reference genes was reliable enough for normalization [33], which is supported by the results of this study.

### 5.2. Reference Gene Validation

**Figure 5:**
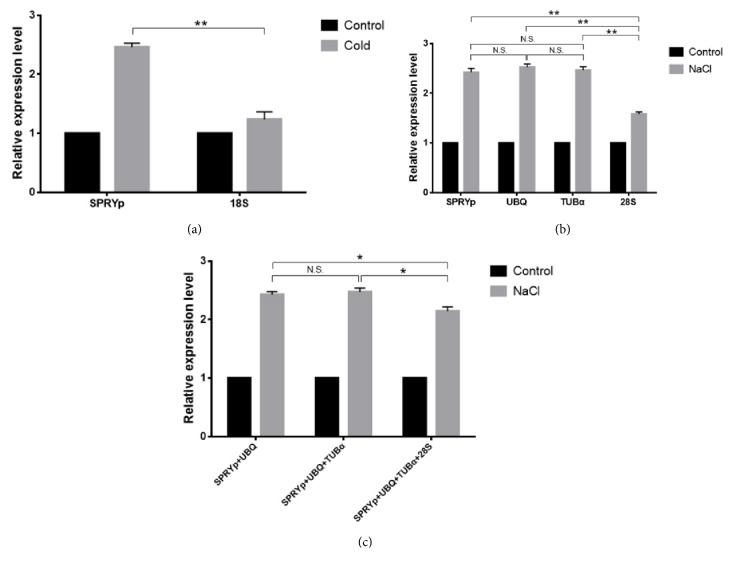
**Validation of reference gene quality. Relative* G6PDH* expression levels were normalized using candidate reference genes under different conditions.** (a) Expression levels were measured in the presence of (a) CuSO_4_ and (b) NaCl and normalized using the most and least stable reference genes.* SPRYp*,* UBQ,* and* TUBα* represent the three in five most stable reference genes and* 28S* the least stable gene in Cold and NaCl. (c) Expression levels were normalized using different combinations of reference genes. Data are displayed as mean ± standard error of the mean. Statistical analyses were performed using Student's t-test to compare two reference genes or combinations of reference genes for normalization. ^*∗*^P<0.05; ^*∗∗*^P<0.01; N.S.: no significant difference.

To evaluate the reliability of the selected reference genes, the relative expression levels of* G6PDH* were calculated. As depicted in Figure 5(a), enhanced expression of* G6PDH* was observed when normalized with the most stable reference gene,* SPRYp*. Meanwhile, when* 28S*, one of the least stable reference genes, was used, a notable reduction in expression was observed. To further evaluate the reliability of the selected reference genes, another stimulus was imposed and the three most stable reference genes were used to analyze the expression of* G6PDH*. The expression of* G6PDH* was enhanced to the same level when normalized with no significant differences between reference genes (Figure 5(b)). However, a significant difference (P<0.01) between reference genes was observed in* G6PDH *expression when using* 28S*, one of the most unstable reference genes. Using geNorm, the optimal number of reference genes for use in normalization was also investigated. While* 28S* was not a suitable reference gene, the accuracy of the results became satisfactory when normalization was performed using* 28S *in combination with other stable genes (Figure 5(c)).

## 6. Discussion

Due to its high sensitivity and specificity, RT-qPCR is now commonly used in many laboratories for high-throughput analysis of gene transcription. Utilizing suitable reference genes is necessary to ensure the reliability and accuracy of the resulting data, as the use of unstable reference genes could yield inaccurate results. Therefore, numerous studies have been conducted to investigate reference gene stability under different conditions [29, 30, 37, 38]. In this present study, the stability of expression of 12 candidate* V. volvacea* reference genes was systematically analyzed using geNorm, NormFinder, and BestKeeper in the presence of salt (NaCl), oxidative (H_2_O_2_), metal (CuSO_4_), acid (pH 4), alkali (pH 9), cold (4°C), and heat stress (42°C), and during different developmental stages. Based on their differential stability, it was found different genes were optimal as references under different conditions.

In this study, the 12 reference genes were first cloned from cDNA template, although PCR was also conducted using genomic DNA as template. As shown in Fig.  S1, the primers were specific and the PCR products from different templates of different lengths. Primer pair specificity was also confirmed by melting curve analysis (Fig.  S2), while amplification efficiency was calculated based on the slopes of the standard curves. The R2 >0.99 and E-values ranged from 92.34 to 109.23% (Table 1 and Fig.  S3), where there was a good linear relationship based on the standard curves and acceptable PCR conditions.

The expression levels of the selected genes were also investigated and the mean Cps are listed in Figure 1. The average expression levels ranged from 9.37 to 29.56, consistent with previous studies [29, 38]. Because moderate expression levels (e.g., Cp of 15 to 30) yield accurate normalization [39], the genes selected in this study were found to be sufficient for experimental needs. Low Cps correspond with high expression levels; therefore, some candidate genes in this study were abundantly distributed in* V. volvacea*. For instance,* UBQ* had a mean Cp value of 19 in* V. volvacea*, but a Cp of up to 27 in* Ganoderma Lucidum *[40, 41]. A narrow distribution range indicates low variability. Therefore, the variation in Cps observed in this study indicates* CYP* is the most optimal reference gene and* MAPK* is the least. However, these results are somewhat inconsistent with those from geNorm and NormFinder (Figure 2 and Table 2). Based on the differences in the stability and expression levels of the candidate reference genes, stability and expression analyses using different methods need to be combined.

To increase accuracy when analyzing candidate gene stability, three Excel-based programs were used as previously described [29, 33–35]. Because different types of software have distinct methods of ranking candidate gene stability and there might be differences in results, at least two methods had to be used to analyze the data. In addition, because reference gene expression stability differs under different conditions, gene expression was assessed in the presence of salt, oxidative, metal, acid-base, and temperature stresses and during different developmental stages. The treatments conducted in the study included nearly all used in similar studies and, therefore, this present study was a systematic assessment of gene stability [9, 14, 29, 42].

According to the geNorm analysis,* TUBα* and* SPRYp* were the two most stable reference genes for all samples and conditions, which is consistent with the NormFinder, but not BestKeeper, results. For BestKeeper,* CYP* and* L-asp *had lower CVs and were the most stable reference genes. This may be because the geNorm and NormFinder analyses performed calculations of stability in a similar manner, while BestKeeper used CV ± SD to rank stability. This phenomenon was also reported by Zhao and Tian in their studies [14, 30]. However, there tended to be consistency when comparing the five most stable reference genes. For example, for NaCl-induced stress, geNorm, NormFinder, and BestKeeper analyses found* UBQ*>* CYP*>* SPRYp*>* TUBα*>* L-asp*,* UBQ*> S*PRYp*>* CYP*>* L-asp*>* TUBα,* and* SPRYp*>* UBQ*>* MAPK*>* L-asp*>* MSF1*, respectively. Furthermore, when normalized using* SPRYp*,* UBQ*, and* TUBα*, there were no significance differences in* G6PDH *expression (Figure 5(b)). Therefore, predicting reference gene stability using three types of software was sufficient and is a good strategy for selecting reference genes for normalization [43–45]. For example, when the three types of analyses were combined,* SPRYp*,* TUBα*,* MSF 1*,* CYP,* and* L-asp* were the most stable reference genes under the different conditions and were easily at the top of the lists in Figures 2 and 3 and Table 2, where one of these was the optimal reference gene in at least one condition. However, the candidate genes with low stability could also be used for normalization. For example, while* GAPDH* ranked nearly last among the candidate genes, it had a low CV and high expression under cold stress, making it a satisfactory reference under this specific condition. There have also been numerous studies indicating that* GAPDH* is among the most stably expressed genes and is usually used to analyze gene expression [46–49]. Overall, experimental conditions and expression abundance have equal importance when choosing a suitable reference gene.

To the best of our knowledge, this present study was the second to survey* V. volvacea *reference gene stability and provides a basis for further exploration of metabolism and regulation in response to environmental stresses. This study focused on different developmental stages and abiotic stresses (NaCl, CuSO_4_, H_2_O_2_, HCl, NaOH, heat, and cold), while Tao et al. [29] performed the first study on* V. volvacea *internal control genes for different strains, fruiting body developmental stages, and growth stages. Interestingly, the results of these two studies were very similar as* SPRYp*,* TUBα*,* CYP*,* L-asp*, and* MSF1* were the most stable reference genes in this present study, while* SPRYp*,* Ras*,* Vps 26*, and* ACTB* were most stable in Tao's study.* Ras* and* Vps 26* were not included in the 12 candidate genes we selected, but* L-asp*,* TUBα,* and* MSF1, *which were assessed in this study, ranked among the top in Tao's study, supporting our results. Conversely,* ACTB* was among the least stable reference genes in our study, which is inconsistent with Tao et al. [29]. This may be a result of different experimental conditions, because no reference gene was universally stable. Ultimately, the choice of reference gene will depend on the specific set of experiments, to which our efforts are complementary.

To determine how many reference genes are needed for accurate analysis, “pairwise variation (V)” was calculated in geNorm. A V score of 0.15 was used as a cut-off according to the manufacturer's instructions, below which the inclusion of an additional reference gene was not required [33]. Based on this, the optimal numbers of reference genes were calculated and are listed in Figure 3. When analyzing the 9 experimental groups, 7 had a V score <0.15, indicating there was no need for the use of a third reference gene. This is consistent with work by Zhao et al. [14], who saw no notable differences when two or three reference genes were used for normalization, and Ma et al. [9], who evaluated different combinations of reference genes for normalization. However, when the V score >0.15, an additional reference gene is recommended. As previously shown, when one of least stably expressed reference genes,* ACTB, *was combined with other stable genes, the results appeared credible [50, 51]. This also indicates that the proposed V of 0.15 value should not be considered a strict cut-off [33], which is in line with several reports that used higher V values [39, 52].

## 7. Conclusions

When characterizing gene expression, the most commonly used method is RT-qPCR, where a suitable reference gene is necessary for normalization of results. In this present study, 12 candidate reference genes in* V. volvacea* were investigated to determine the most stably expressed under different conditions. Analysis of gene expression stability using geNorm, NormFinder, and BestKepper revealed that* SPRYp*,* TUBα*,* CYP*,* L-asp*, and* MSF1* were the most stably expressed reference genes and were optimal for normalization (Table 3). By contrast,* 18S* and* 28S* were the least stably expressed genes. The optimal number of reference genes for normalization was also calculated based on pairwise variation (Vn/Vn+1) using geNorm and it was found the two most stable reference genes were sufficient for normalization under most conditions. Since gene expression varies in different experiment conditions, this study is the first survey of reference gene stability and providing a basis for further research in* V. volvacea*, it also provides guidelines for obtaining more accurate RT-qPCR results for other edible fungal species.

## Figures and Tables

**Table 1:** Candidate reference genes evaluated for expression stability in *V. volvacea.*

Gene name	Description	Accession	Primer sequence: forward/reverse(5′-3′)	Length (bp)	PCR efficiency	R^2^
ACTB	beta-actin	KF528321	TATCGATAATGGCTCCGGCATGTGC/ATACCACGCTTGGATTGGGCCTCAT	165	116.629	0.998
CYP	cyclophilin	KF528322	AGAATGGCTTTGGATACAAGGGGTC/CCTGAAGTTCTCATCTGCGAATCTCTC	140	114.960	0.998
GAPDH	glyceraldehyde-3-phosphate dehydrogenase	KF528323	GATGCTTACGATCCCAAGTACACCG/CTACGACCACCACGCCAATCTTT	191	112.199	1
TUB*α*	alpha-tubulin	KF528325	GAGCCCAATGTTATCGATGAAGTGC/GTTCTTTGCCAATTGTGTAGTGCCC	130	110.336	0.999
TUB*β*1	beta-tubulin 1	KF525326	GTTGATTTGGAGCCTGGAACTATGG/TCCTTCCGTATAGTGTCCTTTTGCC	132	128.813	0.997
UBQ	ubiquitin	KF528328	CAATCACCTTGGAAGTCGAGTCGTC/CTGGATGTTGTAGTCGGAAAGGGTG	152	107.601	1
MSF1	MSF1-domain-containing protein	KF528329	TCTGTCGACCCCACAACTGGCATAA/TCTGTGTAGCTGGGTCGACGAATGA	145	111.670	0.998
SPRYp	SPRY domain protein	KF528330	GCATTCTTCTTGATGTCGGTGGTCG/AACCCTGAAGTGTTGGATGCTCTGG	130	115.097	0.997
Lasp	L-asparaginase	KF528333	GTCACGTCAAGCCTCAAACCAAAAC/ATCGAATAGACTTCATACCACCTCCCC	157	109.315	0.997
MAPK	mitogen-activated protein kinase	FJ906769	TCCGAACACAAGACCTATCCGACGA/ACAGTTGGCGTTCAGGGAGCAGATT	163	111.201	0.999
18S	18S ribosomal RNA	∖	CCGACACGGGGAGGTAGTGACAATAA/CGCTATTGGAGCTGGAATTACCGC	149	97.636	0.998
28S	28S ribosomal RNA	∖	GAATGCAGCTCAAAATGGGGTGG/GCGACTGACTTCAAGCGTTTCCCT	160	111.048	0.999

**Table 2:** Expression stability of 12 *V. volvacea* reference genes as calculated by NormFinder.

Rank	NaCl	CuSO_4_	H_2_O_2_	Heat	Cold	pH 4	pH 9	Developmental Stage	Total
1	UBQ	UBQ	SPRYp	UBQ	MSF1	MSF1	18S	L-asp	SPRYp
	0.244	0.105	0.164	0.033	0.070	0.132	0.029	0.287	0.354
2	SPRYp	TUB*α*	TUB*β*1	MSF1	SPRYp	TUB*α*	L-asp	TUB*β*1	TUB*α*
	0.253	0.18	0.269	0.083	0.072	0.226	0.139	0.288	0.373
3	CYP	SPRYp	ACTB	CYP	TUB*α*	TUB*β*1	CYP	MAPK	MSF1
	0.425	0.205	0.367	0.304	0.104	0.253	0.250	0.315	0.485
4	L-asp	L-asp	TUB*α*	TUB*β*1	ACTB	ACTB	UBQ	TUB*α*	L-asp
	0.524	0.288	0.403	0.399	0.365	0.365	0.267	0.316	0.553
5	TUB*α*	TUB*β*1	UBQ	ACTB	L-asp	MAPK	MSF1	GAPDH	CYP
	0.534	0.301	0.467	0.410	0.400	0.379	0.297	0.323	0.557
6	18S	ACTB	MSF1	TUB*α*	UBQ	SPRYp	MAPK	18S	GAPDH
	0.674	0.396	0.539	0.439	0.450	0.390	0.319	0.337	0.752
7	ACTB	MSF1	L-asp	SPRYp	CYP	GAPDH	TUB*α*	SPRYp	MAPK
	0.777	0.623	0.615	0.497	0.459	0.436	0.320	0.417	0.826
8	GAPDH	18S	MAPK	GAPDH	28S	28S	TUB*β*1	MSF1	UBQ
	0.803	0.669	0.645	0.637	0.550	0.473	0.365	0.423	0.831
9	MSF1	GAPDH	GAPDH	L-asp	MAPK	CYP	SPRYp	CYP	TUB*β*1
	0.872	0.805	0.853	0.671	0.625	0.480	0.498	0.455	0.838
10	MAPK	CYP	18S	18S	GAPDH	18S	GAPDH	28S	28S
	0.945	0.816	0.872	0.883	0.902	0.490	0.601	0.577	0.843
11	TUB*β*1	MAPK	CYP	28S	TUB*β*1	UBQ	28S	UBQ	18S
	1.221	0.843	0.899	1.142	1.315	0.544	0.769	1.237	1.041
12	28S	28S	28S	MAPK	18S	L-asp	ACTB	ACTB	ACTB
	1.246	0.902	0.979	1.468	1.722	0.661	1.025	1.702	1.207

**Table 3:** Expression stability ranking of the 12 candidate reference genes.

**Method**	**1**	**2**	**3**	**4**	**5**	**6**	**7**	**8**	**9**	**10**	**11**	**12**
**RANKING ORDER UNDER NaCl STRESS (BETTER-GOOD-AVERAGE)**												
**geNorm**	CYP		SPRYp	TUB*α*	L-asp	GAPDH	ACTB	MSF1	18S	MAPK	28S	TUB*β*1
	UBQ											
**NormFinder**	UBQ	SPRYp	CYP	L-asp	TUB*α*	18S	ACTB	GAPDH	MSF1	MAPK	TUB*β*1	28S
**BestKeeper**	SPRYp	UBQ	MAPK	L-asp	MSF1	CYP	TUB*α*	ACTB	TUB*β*1	GADPH	18S	28S
**Comprehensive Ranking**	UBQ	SPRYp	CYP	L-asp	TUB*α*	MAPK	MSF1	ACTB	GAPDH	18S	TUB*β*1	28S
**RANKING ORDER UNDER H**_**2**_ **O**_**2**_ ** STRESS (BETTER-GOOD-AVERAGE)**												
**geNorm**	TUB*α*		SPRYp	ACTB	UBQ	MSF1	MAPK	L-asp	GAPDH	CYP	18S	28S
	TUB*β*1											
**NormFinder**	SPRYp	TUB*β*1	ACTB	TUB*α*	UBQ	MSF1	L-asp	MAPK	GAPDH	18S	CYP	28S
**BestKeeper**	MAPK	MSF1	UBQ	SPRYp	TUB*β*1	L-asp	ACTB	TUB*α*	CYP	GADPH	28S	18S
**Comprehensive Ranking**	TUB*β*1	SPRYp	TUB*α*	MAPK	MSF1	UBQ	ACTB	L-asp	GAPDH	CYP	18S	28S
**RANKING ORDER UNDER CuSO**_**4**_ ** STRESS (BETTER-GOOD-AVERAGE)**												
**geNorm**	TUB*α*		TUB*β*1	ACTB	L-asp	SPRYp	CYP	GAPDH	MSF1	18S	MAPK	28S
	UBQ											
**NormFinder**	UBQ	TUB*α*	SPRYp	L-asp	TUB*β*1	ACTB	MSF1	18S	GAPDH	CYP	MAPK	28S
**BestKeeper**	SPRYp	L-asp	UBQ	TUB*β*1	TUB*α*	MSF1	MAPK	ACTB	CYP	GADPH	18S	28S
**Comprehensive Ranking**	UBQ	TUB*α*	SPRYp	L-asp	TUB*β*1	ACTB	MSF1	CYP	GAPDH	MAPK	18S	28S
**RANKING ORDER UNDER COLD STRESS (BETTER-GOOD-AVERAGE)**												
**geNorm**	MSF1		TUB*α*	ACTB	L-asp	UBQ	CYP	28S	GAPDH	MAPK	TUB*β*1	18S
	SPRYp											
**NormFinder**	MSF1	SPRYp	TUB*α*	ACTB	L-asp	UBQ	CYP	28S	MAPK	GAPDH	TUB*β*1	18S
**BestKeeper**	GADPH	UBQ	L-asp	CYP	MSF1	SPRYp	TUB*α*	MAPK	ACTB	28S	TUB*β*1	18S
**Comprehensive Ranking**	MSF1	SPRYp	TUB*α*	UBQ	L-asp	GAPDH	ACTB	CY11P	28S	MAPK	TUB*β*1	18S
**RANKING ORDER UNDER HOT STRESS (BETTER-GOOD-AVERAGE)**												
**geNorm**	UBQ		CYP	ACTB	TUB*β*1	TUB*α*	L-asp	GAPDH	SPRYp	18S	28S	MAPK
	MSF1											
**NormFinder**	UBQ	MSF1	CYP	TUB*β*1	ACTB	TUB*α*	SPRYp	GAPDH	L-asp	18S	28S	MAPK
**BestKeeper**	MSF1	TUB*α*	L-asp	GADPH	SPRYp	TUB*β*1	CYP	18S	ACTB	UBQ	MAPK	28S
**Comprehensive Ranking**	MSF1	UBQ	CYP	TUB*α*	TUB*β*1	ACTB	L-asp	GAPDH	SPRYp	18S	28S	MAPK
**RANKING ORDER UNDER ACID STRESS (BETTER-GOOD-AVERAGE)**												
**geNorm**	SPRYp		MSF1	TUB*β*1	TUB*α*	28S	18S	GAPDH	ACTB	CYP	UBQ	L-asp
	MAPK											
**NormFinder**	MSF1	TUB*α*	TUB*β*1	ACTB	MAPK	SPRYp	GAPDH	28S	CYP	18S	UBQ	L-asp
**BestKeeper**	TUB*β*1	MAPK	SPRYp	MSF1	TUB*α*	L-asp	CYP	UBQ	ACTB	GADPH	28S	18S
**Comprehensive Ranking**	MAPK	MSF1	TUB*β*1	SPRYp	TUB*α*	ACTB7	28S	GAPDH	CYP	18S	L-asp	UBQ
**RANKING ORDER UNDER ALKALI STRESS (BETTER-GOOD-AVERAGE)**												
**geNorm**	MSF1		18S	L-asp	CYP	UBQ	TUB*α*	TUB*β*1	SPRYp	GAPDH	28S	ACTB
	MAPK											
**NormFinder**	18S	L-asp	CYP	UBQ	MSF1	MAPK	TUB*α*	TUB*β*1	SPRYp	GAPDH	28S	ACTB
**BestKeeper**	L-asp	MSF1	MAPK	SPRYp	GADPH	CYP	TUB*α*	UBQ	TUB*β*1	18S	ACTB	28S
**Comprehensive Ranking**	L-asp	MSF1	MAPK	18S	CYP	UBQ	SPRYp	TUB*α*	GAPDH	TUB*β*1	28S	ACTB
**RANKING ORDER UNDER DIFFERENT STAGES (BETTER-GOOD-AVERAGE)**												
**geNorm**	TUB*β*1		TUB*α*	L-asp	18S	GAPDH	SPRYp	MSF1	CYP	28S	UBQ	ACTB
	MAPK											
**NormFinder**	L-asp	TUB*β*1	MAPK	TUB*α*	GAPDH	18S	SPRYp	MSF1	CYP	28S	UBQ	ACTB
**BestKeeper**	SPRYp	GADPH	L-asp	CYP	UBQ	MAPK	MSF1	TUB*α*	TUB*β*1	18S	28S	ACTB
**Comprehensive Ranking**	L-asp	TUB*β*1	MAPK	SPRYp	GAPDH	TUB*α*	18S	CYP	MSF1	UBQ4	28S	ACTB
**RANKING ORDER UNDER ALL SAMPLES (BETTER-GOOD-AVERAGE)**												
**geNorm**	TUB*α*		L-asp	CYP	GAPDH	MSF1	UBQ	28S	MAPK	TUB*β*1	18S	ACTB
	SPRYp											
**NormFinder**	SPRYp	TUB*α*	MSF1	L-asp	CYP	GAPDH	MAPK	UBQ	TUB*β*1	28S	18S	ACTB
**BestKeeper**	CYP	L-asp	MSF1	MAPK	GADPH	ACTB	SPRYp	TUB*β*1	TUB*α*	UBQ	18S	28S
**Comprehensive Ranking**	SPRYp	TUB*α*	CYP	L-asp	MSF1	GAPDH	MAPK	UBQ	TUB*β*1	ACTB	28S	18S

## Data Availability

All the figures and tables used to support the findings of this study are included within the article and supplementary information files.

## Supplementary Materials

Supplementary Materials
** Fig. S1:** agarose gel (1%) electrophoresis of the 12 candidate reference genes.** Fig. S2:** melt curves for the 12 candidate reference genes.** Fig. S3:** standard curves for the 12 candidate reference genes.** Table S1:** raw Cp values for* V*. * volvacea*.
