# Supplementary material for: Selection and Evaluation of Appropriate Reference Genes for RT-qPCR Normalization of Volvariella volvacea Gene Expression under Different Conditions
Source: Biomed Res Int. 2018 Jul 9;2018:6125706. doi: 10.1155/2018/6125706 (PMC6069580; doi:10.1155/2018/6125706)
Supplement: Supplementary Materials — Fig. S1: agarose gel (1%) electrophoresis of the 12 candidate reference genes. Fig. S2: melt curves for the 12 candidate reference genes. Fig. S3: standard curves for the 12 candidate reference genes. Table S1: raw Cp values for V. volvacea. [file 6125706.f1.pdf]

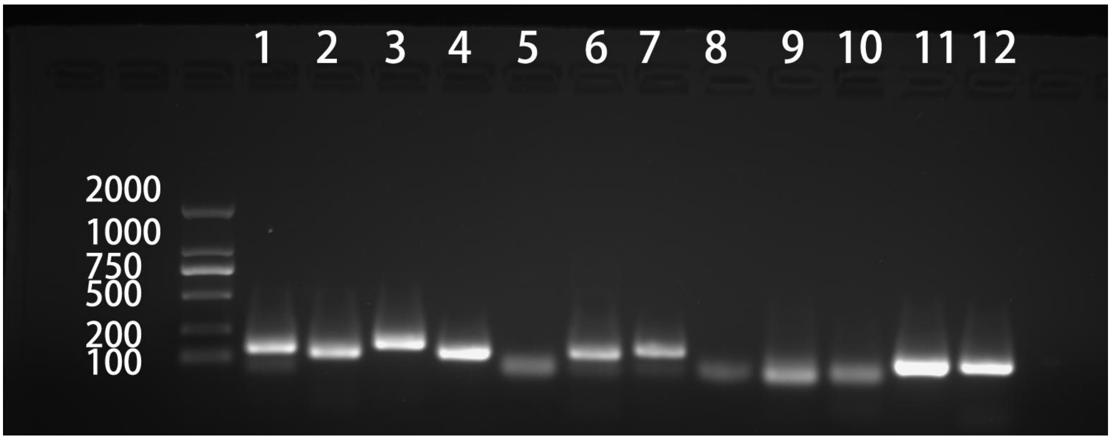

Fig. S1. Agarose gel (1%) electrophoresis of the 12 candidate reference genes. PCR products for *ACTB, CYP, GAPDH, TUBα, TUBβ1, UBQ, MSF1, SPRYp, L-asp, MAPK, 18S, and 28S* are presented in lanes 1-12, respectively.

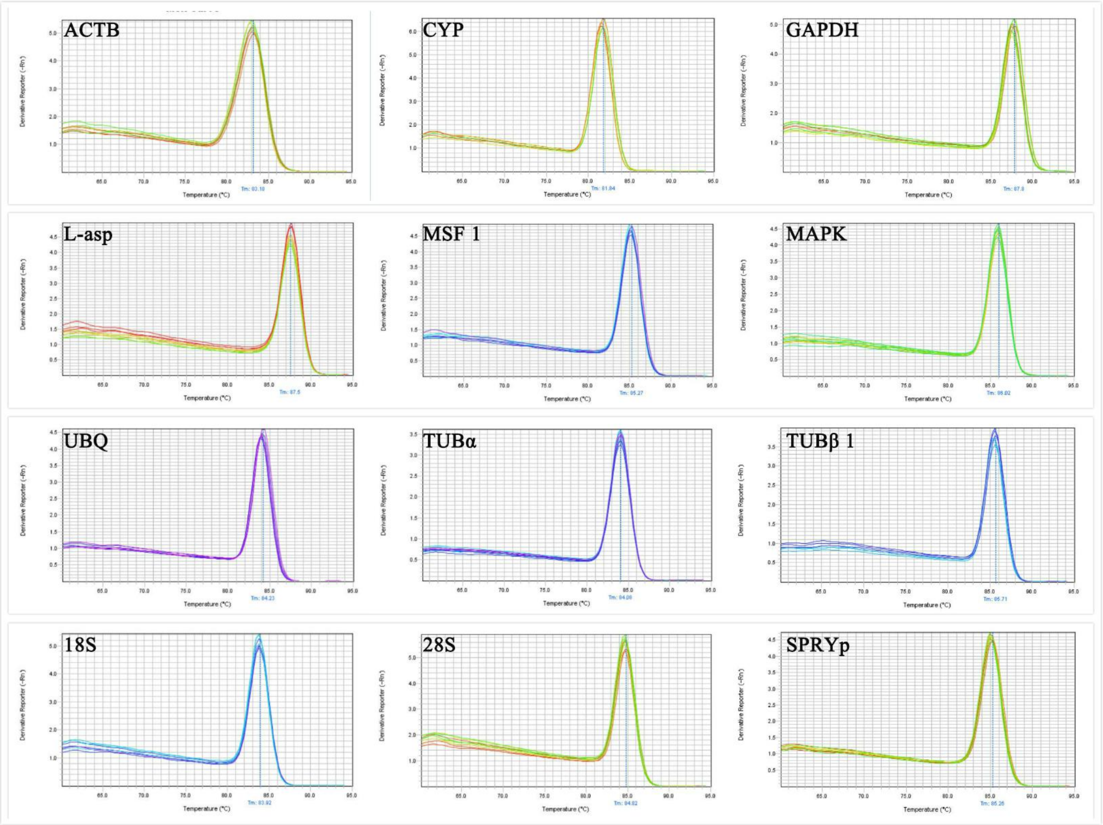

Fig. S2. Melt curves for the 12 candidate reference genes

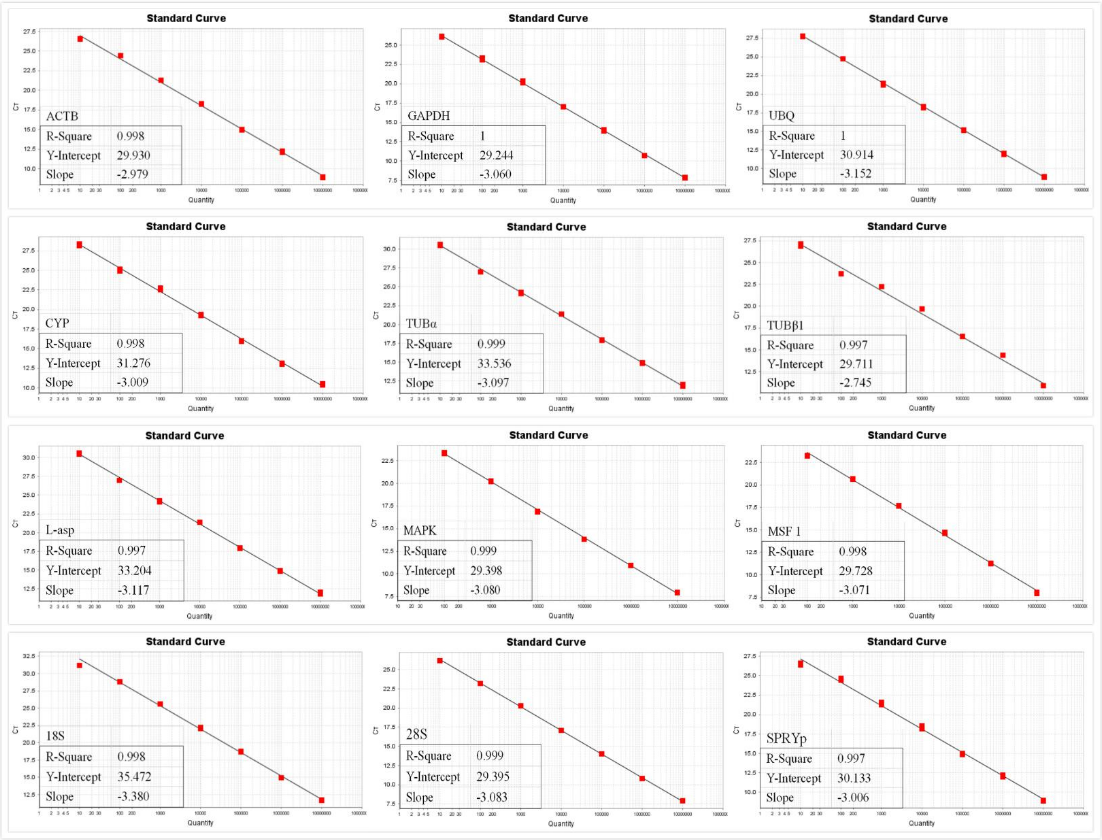

Fig. S3. Standard curves for the 12 candidate reference genes

Table S1. Raw Cp values for *V. volvacea*

Cps for *V. volvacea* mycelia exposed to NaCl, $CuSO_4$, H_2O_2, HCl, NaOH, heat, and cold, as well as *V. volvacea* samples from different developmental stages.

	ACTB	CYP	GAPDH	TUBα	UBQ	MSF1	SPRYp	L-asp	TUBβ1	MAPK	18S	28S
	21.91	20.07	21.03	19.17	21.99	28.17	24.30	28.22	24.05	28.70	8.12	12.47
	20.36	19.71	21.44	19.00	21.90	28.48	24.35	28.06	23.96	28.78	8.07	11.31
	21.03	19.55	20.93	18.97	21.55	28.09	24.36	28.36	23.85	28.77	8.03	11.31
	20.04	19.80	21.75	19.65	21.91	29.75	25.34	29.79	23.42	28.17	9.59	10.85
**NaCl**	21.06	19.55	22.40	20.31	22.28	29.58	25.09	29.83	22.91	28.30	9.53	10.89
	20.22	19.84	21.97	20.46	21.93	29.58	24.95	30.01	19.97	28.71	9.52	10.95
	23.10	21.49	24.42	21.64	23.44	29.71	26.10	30.08	21.71	26.75	7.92	8.80
	23.15	21.40	24.34	21.34	23.24	32.50	25.98	31.03	21.57	26.77	7.82	8.78
	23.26	21.70	24.16	21.46	23.44	27.90	26.16	30.41	21.53	26.67	7.80	8.70

	22.69	23.65	22.82	22.70	21.86	26.49	29.22	32.96	25.30	28.88	8.51	11.30
	21.67	24.00	23.24	22.75	21.64	26.09	28.93	31.58	25.51	28.81	8.44	11.19
	23.02	23.97	22.39	22.57	21.95	25.97	28.68	31.66	25.29	28.58	8.42	11.14
	20.50	21.39	18.48	19.60	21.23	25.93	27.30	28.75	22.83	29.20	7.56	11.44
**H_2O_2**	19.85	21.27	18.58	19.76	21.72	25.88	26.99	29.05	22.63	29.00	7.45	11.51
	19.65	21.17	18.69	19.60	21.72	26.29	26.97	29.03	22.58	28.99	7.43	11.79
	22.02	20.39	20.42	21.63	21.85	25.91	28.78	29.89	24.17	28.95	10.62	13.55
	22.02	20.80	20.45	21.56	22.20	25.89	28.38	29.85	24.20	29.04	10.53	13.61
	21.54	20.66	20.30	21.95	21.72	25.70	28.69	29.74	24.09	28.91	10.49	13.67
	23.99	24.96	22.37	23.36	22.79	24.97	28.04	30.86	26.81	28.55	9.37	10.20
	24.27	24.65	22.43	23.36	22.72	25.00	28.09	30.11	26.83	28.51	9.39	10.35
	24.48	24.66	22.51	23.37	22.74	24.91	28.47	30.28	26.89	28.48	9.32	10.31
	22.91	22.12	21.11	22.37	21.93	25.72	28.68	29.55	25.64	29.37	11.41	11.81
**$CuSO_4$**	23.52	22.07	21.11	22.51	22.01	25.72	28.83	29.83	25.65	29.28	11.21	13.81
	23.66	21.99	21.17	22.52	21.93	25.52	28.73	29.63	25.54	29.64	11.16	12.38
	22.40	23.06	19.46	22.31	21.84	26.57	27.82	30.42	25.96	30.98	9.79	10.74
	22.95	22.89	19.34	22.35	21.91	26.47	27.86	30.49	25.85	30.63	9.72	10.81
	22.50	23.14	19.74	22.26	21.89	26.39	27.83	29.54	25.85	30.35	9.74	10.70
	23.66	23.01	26.61	22.85	16.88	22.62	25.42	32.92	27.73	29.31	9.98	8.06
	23.61	23.69	25.81	24.56	16.78	22.55	25.46	32.76	27.80	29.28	9.91	8.27
	23.81	22.85	25.42	23.59	16.92	22.69	25.82	33.11	27.66	29.30	9.88	7.97
	21.88	21.56	25.53	21.96	15.67	21.78	25.39	30.11	25.49	31.58	9.66	9.52
**Heat**	21.33	21.81	22.86	21.83	15.67	21.75	25.49	29.86	25.44	31.37	9.44	9.39
	21.34	21.96	23.25	21.69	15.65	21.93	25.85	30.11	25.36	31.55	9.25	9.41
	24.58	24.45	25.81	23.44	18.20	23.92	27.65	32.89	28.46	34.36	8.83	7.92
	24.73	24.99	25.89	23.47	18.36	23.91	28.23	32.72	28.13	34.88	8.70	7.86
	24.50	24.35	26.27	23.57	17.92	23.94	28.11	31.62	27.91	34.48	8.68	7.77
	21.28	19.82	21.95	23.33	18.24	25.93	21.53	30.94	21.46	27.91	8.06	8.17
	21.11	19.79	21.96	23.36	18.19	25.93	21.39	29.97	25.42	26.11	7.45	8.56
	20.97	19.66	21.96	23.32	18.11	25.96	21.56	30.04	27.28	27.51	8.62	8.65
	23.61	21.12	21.40	25.84	18.95	27.93	23.47	31.95	26.65	31.03	15.76	9.36
**Cold**	23.51	20.95	21.28	25.90	18.88	27.81	23.56	31.98	27.36	31.29	13.45	9.49
	23.37	20.88	21.27	25.84	18.78	27.83	23.81	31.93	28.62	30.29	14.34	9.67
	21.50	20.74	21.13	24.69	18.32	26.15	22.13	29.98	25.08	29.16	11.64	9.87
	21.92	20.69	21.22	24.69	18.62	26.51	22.15	30.12	24.37	29.54	14.10	10.22
	20.73	21.93	21.55	24.42	18.98	26.15	22.15	30.47	23.84	28.95	13.97	10.33
	22.11	21.67	16.58	21.67	22.94	27.50	28.48	31.29	25.00	27.95	9.37	11.70
	22.20	21.78	17.79	21.78	22.66	27.71	28.16	29.92	25.00	27.91	9.44	11.72
	22.29	21.46	16.55	21.46	22.91	27.85	28.54	31.20	24.88	28.06	9.44	11.71
	24.88	23.81	17.84	22.69	24.96	28.95	28.06	32.39	25.33	28.19	10.00	12.34
**pH 4**	23.86	22.62	18.53	23.57	24.55	28.68	29.12	32.66	25.22	28.04	10.97	12.39
	23.68	22.83	18.34	22.21	24.92	28.19	28.44	33.57	25.84	27.57	10.96	12.47
	24.31	22.01	18.18	22.74	23.33	29.00	29.36	31.47	25.59	28.85	11.91	13.94
	23.95	21.94	18.90	22.63	23.25	28.84	29.70	31.30	25.45	28.72	11.89	13.87

	23.89	21.81	19.48	22.64	23.12	29.11	29.45	31.06	25.39	28.78	11.85	13.99
	19.61	21.41	21.96	24.85	19.43	24.67	27.23	28.19	23.27	28.37	7.94	10.17
	19.89	21.30	21.49	24.93	18.08	24.64	26.01	28.29	23.18	28.52	8.02	9.01
	20.16	21.94	23.16	24.48	19.50	24.41	24.82	28.56	23.07	28.57	8.02	9.36
	22.73	22.63	22.51	25.87	19.49	24.13	26.23	28.66	24.52	27.75	7.86	7.43
**pH 9**	22.69	22.73	21.40	25.86	19.69	24.03	25.86	28.72	24.32	27.85	7.84	7.60
	22.76	22.17	21.01	25.68	19.67	23.92	25.58	28.76	24.20	27.75	7.86	7.32
	23.95	23.05	22.04	26.94	20.64	24.89	26.45	29.22	25.55	28.96	9.16	9.67
	23.89	22.90	21.87	26.86	20.70	24.80	26.39	29.06	25.44	28.77	9.04	9.63
	23.76	22.69	21.89	26.78	20.62	24.92	26.39	29.25	25.40	28.79	9.05	9.94
	25.20	24.87	24.71	20.61	17.67	30.09	25.30	30.93	22.32	27.95	11.01	15.96
	25.10	24.85	24.38	20.38	17.88	30.05	25.38	29.64	22.26	27.99	10.93	15.92
	24.96	24.75	24.34	20.44	17.71	30.12	25.45	29.35	22.13	27.95	10.91	15.88
	21.95	23.37	21.89	15.89	14.95	25.47	21.80	25.63	17.76	22.72	8.09	11.65
	21.77	23.06	21.81	15.90	14.86	25.39	21.73	25.66	17.66	22.70	8.00	11.62
	21.72	22.97	21.76	15.79	14.76	25.30	21.89	25.68	17.53	22.60	7.94	11.47
	22.15	22.95	22.16	16.55	15.22	26.55	23.22	26.65	19.23	24.29	8.15	13.49
**Devel-**	22.17	23.16	23.12	16.47	15.47	26.85	23.46	26.49	19.09	24.15	8.05	13.54
**opme-**	22.85	23.15	22.95	16.46	15.49	26.51	23.47	26.65	19.06	24.13	7.99	13.58
**ntal**	16.53	21.38	20.67	15.62	17.78	24.12	22.67	26.94	16.58	22.30	7.59	13.82
**Stages**	16.33	21.18	20.50	15.67	17.76	24.00	22.59	25.28	16.40	22.32	7.50	12.28
	16.19	21.13	20.47	15.42	17.70	24.06	22.63	25.26	16.39	22.24	7.48	11.89
	16.36	22.31	21.57	16.18	16.84	24.00	22.20	25.64	17.75	22.20	6.96	10.25
	16.01	21.99	21.53	16.24	16.80	23.93	22.20	25.64	17.40	22.15	6.94	10.44
	15.84	21.87	21.46	16.20	16.67	23.91	22.35	25.66	17.32	22.13	6.93	10.28
	17.52	21.53	22.12	16.53	16.99	24.54	23.28	25.80	17.99	23.73	8.63	12.19
	17.34	21.43	21.94	16.45	16.94	24.39	23.27	25.81	17.81	23.84	8.47	12.19
	17.26	21.26	21.91	16.30	16.95	24.39	23.26	25.86	17.74	23.69	8.50	11.84
